# Online Charge Balancing With Active Impedance Monitoring in Programmable Stimulator to Extend Electrode Lifetime

**DOI:** 10.1109/access.2026.3674053

**Published:** 2026-03-13

**Authors:** KANGNI LIU, GUANGZONG CHEN, KEVIN WOEPPEL, X. TRACY CUI, RAJKUMAR KUBENDRAN

**Affiliations:** 1 Department of Electrical and Computer Engineering, University of Pittsburgh, Pittsburgh, PA 15260, USA; 2 Vanish Therapeutics, Pittsburgh, PA 15213, USA; 3 Department of Bioengineering, University of Pittsburgh, Pittsburgh, PA 15260, USA

**Keywords:** Biphasic stimulator, active impedance monitoring, online charge balancing, therapeutic device, peripheral nerve stimulation

## Abstract

Neurostimulation therapies are often applied as an alternative method to pharmaceutical treatment for pain relief. This paper demonstrates a programmable stimulator for analgesic nerve stimulation through invasive electrodes. It provides two modes of operation: constant-current biphasic and capacitor-coupled biphasic. The stimulator is integrated on a 50 × 30 mm Printed Circuit Board (PCB) and can generate up to ±1.6 mA current pulses in steps of 6.3μA. A custom STM32 microcontroller library is used to tune the pulse duration range from 1μs to 10 s and stimulation frequency range between 0.1 Hz to 100 kHz. An Active Impedance Monitoring (AIM) unit in the PCB measures the frequency response from 1 Hz to 10 kHz with 8 samples per decade every 30 minutes. The electrode resistance, tissue resistance and RC time constant of the electrode-tissue system could be calculated from the frequency response curve, generated by an input square wave stimulation current at different frequencies. In different measurements with discrete RC circuit components, the PCB could calculate the impedance values with >90% precision. A novel charge-balancing algorithm implemented on the STM32 microcontroller could match the positive and negative charge within a 5% error. Through online adjustment of stimulation parameters using active impedance monitoring to achieve charge balancing, the proposed programmable stimulation system extends the electrode lifetime for more than 288 million continuous pulses in vitro studies. The in vitro studies clearly demonstrated the efficacy of charge balanced stimulation that enable the electrodes to last around 3 times longer with negligible degradation.

## I. INTRODUCTION

Neurostimulation therapy possesses substantial potential in the treatment of chronic pain and psychiatric disorders. The 2012 National Health Interview Survey reports that approximately 11.2% of the adult population in the United States experiences chronic pain on a daily basis [1], for which various treatments are available, including tricyclic antidepressants, serotonin, repetitive transcranial magnetic stimulation, Gabapentin, and psychological therapy [2]. However, patients often experience limited relief due to the modest effectiveness of these treatments, potential side effects, and therapy restrictions [3], [4], [5]. Consequently, there is a demand for alternative treatment approaches. For individuals with unresolved chronic pain post-surgery or those not suitable for surgery, electrical stimulation and neuromodulation offer considerable relief. Currently, neurostimulation therapy provides pulse control with a precision of nano amperes (nA) [6], and pulse amplitude adjustment in real-time [7]. This capability fulfills the essential requirement for precise and targeted interventions for effective treatments. Techniques such as spinal cord stimulation (SCS) [8], [9], vagus nerve stimulation (VNS) [10], and peripheral nerve stimulation (PNS) [11], [12] have shown considerable effectiveness in mitigating symptoms associated with Parkinson’s disease, chronic pain, epilepsy, and various other neurological conditions [13], [14].

There are two primary stimulation methods used for therapy: voltage-controlled stimulation (VCS) and current-controlled stimulation (CCS). VCS applies voltage signals to the tissue. However, the charge delivered to the tissue can fluctuate depending on the electrode material, design, impedance, and attachment status. As a result, maintaining a charge balance in VCS mode is challenging, which is vital for patients. A persistent residual charge can harm both the electrodes and the target tissue over time.

Conversely, the impact of CCS remains uninfluenced by electrode-tissue impedance when using current-controlled stimulation. In this context, CCS emerges as the most effective stimulation method for chronic pain management. By delivering a current stimulus via the spinal cord or nerve, pain perception can be alleviated. The crucial aspect is that CCS’s effect is not hindered by impedance change, enabling optimal therapeutic outcomes. Thus, CCS is widely utilized in neuromodulation. Unlike pharmaceutical treatments, this approach presents a low risk of overdose and abuse. Furthermore, sustained pain relief can be maintained even after the cessation of stimulation [12].

The four modes of CCS are monopolar monophasic, monopolar biphasic, bipolar monophasic, and bipolar biphasic. In monopolar stimulation, a single electrode is used to deliver the current, with a typically utilized screw as a reference point. In contrast, bipolar stimulation involves two electrodes positioned closely in the target area. Typically, the working electrode is applied with monophasic stimulation for its high efficiency. However, greater negative overpotentials are reached during monophasic pulsing than with biphasic pulsing. This process will cause irreversible reactions, such as oxygen reduction and electrode corrosion. Thus, monophasic pulses are not used in long-term stimulation to avoid electrode damage. For biphasic stimulation, if the second phase starts immediately after the first, it may reverse some of the desired physiological effects of the first phase [15]. By introducing an open-circuit interphase delay between the stimulating and reversal phases, the electrode could fully rest from the stimulation and the neuronal response could be shaped.

**FIGURE 1.**
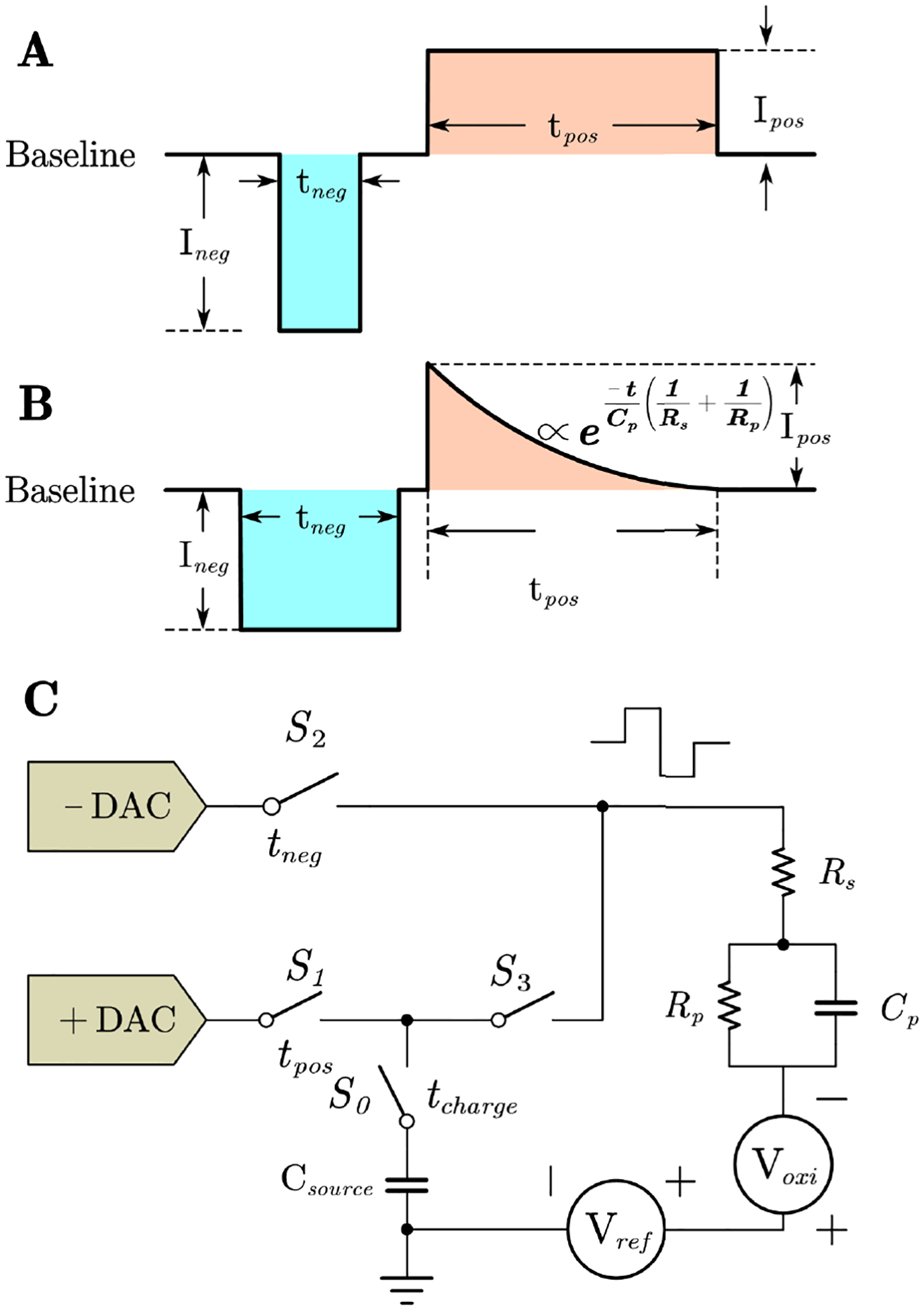
Stimulation current waveform in two modes. The positive pulse duration is $t_{pos}$, and the amplitude is $I_{pos}$. The negative pulse duration is $t_{neg}$, and the amplitude is $I_{neg}$. The total period of the stimulation could be set up to 10 s. **A** Biphasic mode current waveform. **B** Capacitor-coupled biphasic mode current mode. The exponential decay current is achieved by discharging the capacitor $C_{\text{source}}$. **C** Circuit diagram of programmable stimulator with DACs, switches and electrode model. In biphasic mode without capacitor coupling, the switch $S_{3}$ is closed and $S_{0}$ is open all the time. In biphasic mode with capacitor coupling, the capacitor $C_{\text{source}}$ is charged during negative phase and then discharged in the positive phase resulting in an exponentially decaying current.

Thus, the most widely used stimulation waveform is the biphasic wave, which applies two square waves in different polarities, as shown in Fig. 1 (a). The capacitor-coupled waveform shows another possibility of stimulation. It consists of 2 phases. The first phase is a negative square wave. In the second phase, the coupled capacitor discharges and forms a smooth exponential decay wave, as shown in Fig. 1 (b).

The anodic break may be prevented by using stimulating pulses with slowly decaying exponential phases instead of abrupt terminations [16], [17]. It has been proved that the more rapidly the charge is injected during the negative phase, the less likely that tissue damage will occur [18]. Compared to the square wave, the rapid exponential decay wave performs faster in electrode potential balance. Thus it is more friendly to the stimulated tissue. Also, monophasic stimulation enabled selective stimulation of fibers over cells or cells over fibers, respectively. However, when the asymmetrical charge-balancing stimulus phase was incorporated, selectivity was greatly diminished [19]. The capacitor-coupled biphasic waveforms provided better selectivity.

It is noticed that the charge balance calculation is more complicated in capacitor-coupled biphasic stimulation. In typical biphasic stimulation, the current is a constant value in both phases. The charge can be calculated by the equation, Q = It. The charge is proportional to time, and it is easy to control. However, the discharge equation of the coupled capacitor is an exponential decay curve. To match the charge with the exponential wave, the temporal resolution of the negative phase should be very small. For example, in Fig. 1(b), considering the positive phase $I_{\text{pos}}$ is 1.6 mA square wave and $t_{pos}$ is 2 ms. The time constant τ is 8 ms. If $I_{neg}$ is 1 mA, $t_{neg}$ should be 882μs to make the charge balanced. Capacitor-coupled biphasic requires high-accuracy control for the pulse duration.

An electrical nerve stimulator is a biomedical device designed to deliver electrical currents into biological tissue through an electrode, to modulate the activity of local neuronal populations at the target site. These stimulators are widely used in therapeutic interventions [20], [21]. Present commercial nerve stimulators demonstrate significant potential in achieving precise current control and compact size. However, a limited number of stimulators recognize the evolving trends in electrode behavior. The standard of care for traditional SCS necessitates invasive surgical interventions and presents a complication rate ranging from 30–40% [22], [23]. Complications arising from device migration within tissues and lead fractures have been identified. Predominantly, available electrodes consist of metal [24], [25], [26]. These electrodes corrode when stimulated above the charge injection limit or under unbalanced charge injection. Electrode-tissue interface impedance can fluctuate *in vivo* due to injury and foreign body responses. Upon damage to the electrodes, there is a significant increase in impedance. For the electrode-tissue system, such variations in electrode impedance result in an amplified high-frequency response.

**FIGURE 2.**
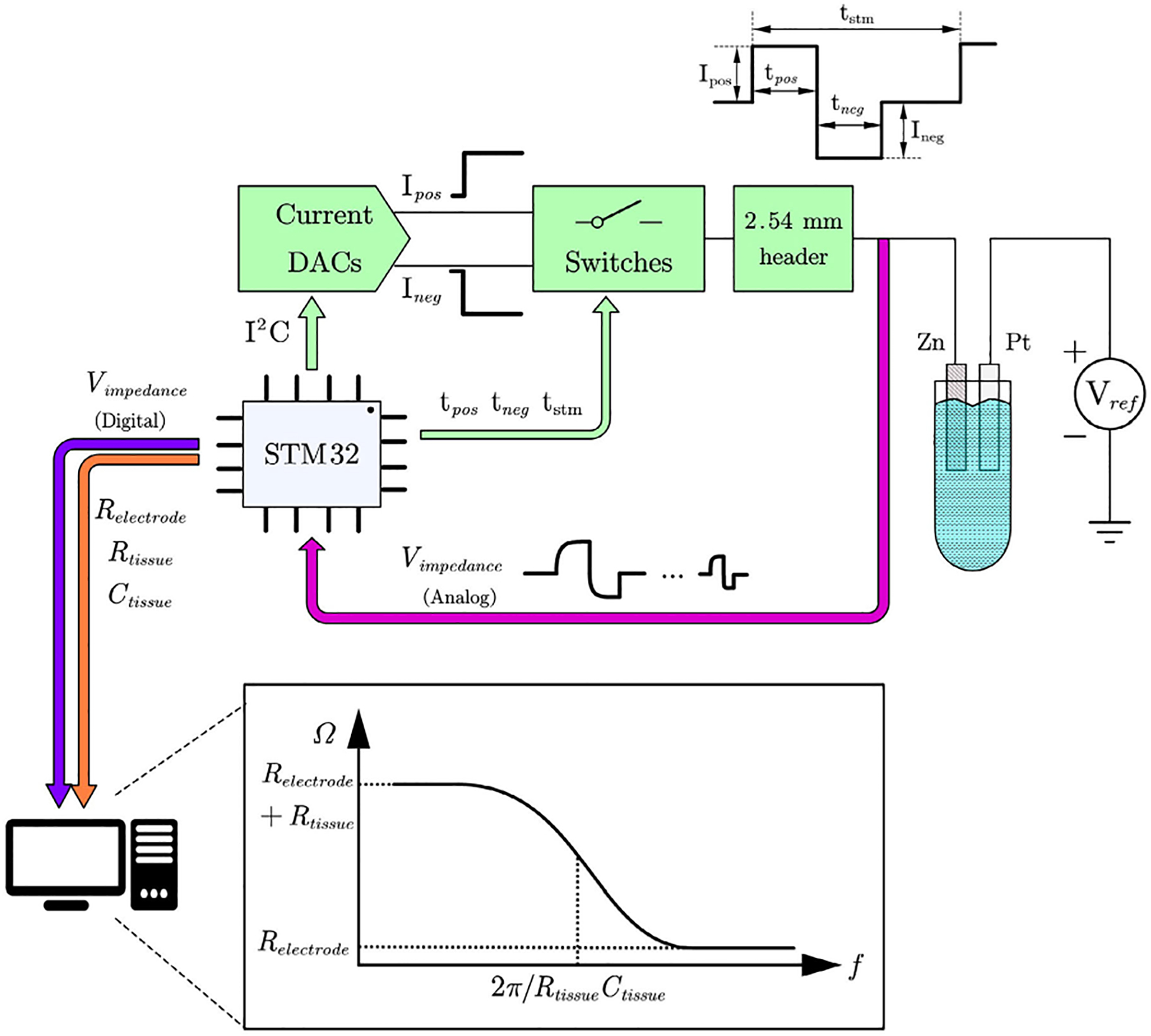
Block diagram of the stimulation system. The STM32 microcontroller decides the current pulse amplitude and length. The current pulses are sent to the zinc electrode, which is soaked in sterile saline solution. The voltage across the electrode will be measured by STM32 ADC. The STM32 calculates the impedance value for the electrode-tissue system and sends it to the computer (optional) to plot the frequency response. STM32 adjusts the pulse length according to the impedance calculation result to perform charge balancing. The stimulation current is feed directly to the ADC through 2.54 mm headers.

This study introduces a online charge balancing PNS system capable of tracking electrode-tissue interface impedance and adjusting charge injection accordingly, as shown in Fig. 2. The VDD and VSS for this system is 3.3V and 0V respectively. The reference voltage $V_{\text{ref}}$ is a 3.3V constant voltage. It delivers a maximum current of ±1.6 mA. By introducing high accuracy of charge calculation in the system, the risk of electrode corrosion and tissue injury can be minimized at the same time. To facilitate the measurement of electrode impedance and extend the potential applications of neurostimulation therapy, a sequence of square current pulses is systematically administered to the electrode. The frequency of these current pulses varies between 1 Hz and 10 kHz. Furthermore, colored LED lights are utilized to signify trends in electrode impedance changes.

## II. MATERIAL AND METHODS

This paper is an extended work based on [27]. Several significant innovations and improvements have made compared to [27]. 1. Impedance monitoring is integrated on the PCB, instead of measuring through a bulky commercial equipment like Autolab, used in [27]. 2. Designed and implemented a charge balancing algorithm for the capacitor-coupled biphasic stimulation. While in [27], the parameters for charge balancing were calculated manually before the program setup. 3. Performed several studies with discrete components and multiple *in vitro* experiments. Only 1 *in vitro* study is shown in [27].

### A. SYSTEM DESIGN OVERVIEW

The programmable stimulator with AIM unit is constructed on a printed circuit board (PCB) measuring 50 mm by 30 mm, as depicted in Fig. 4. The current pulse parameters and impedance measurement range are designed for a bioresorbable metal electrode. The details of the electrode will be presented in the Results section. The size and weight of the stimulator is designed for long term animal study experiments. With a mass of 50 grams, it is suitably lightweight for experimentation on small animals. The system consists of a voltage regulator, four 8-bit sink/source current digital-to-analog converters (DACs), eight analog switches, two LED lights, and an STM32 microcontroller. The DAC output is directly connected to the electrode through a 2.54 mm header. The STM32 microcontroller is disconnected from the computer after programming and can work independently under battery power.

The entire system operates at 3.3 V DC supply voltage. The voltage regulator accommodates an input voltage range from 4.2 V to 9 V, that can be provided through batteries or an external supply. Testing with 100 Hz 10% duty cycle biphasic mode stimulation, the system is capable of functioning continuously for more than 72 hours when powered by two 3 V coin cell batteries. In this case, the STM32 microcontroller system clock is 1 MHz. The coin battery is CR2450 and the capacity is 620 mAh and the average current consumption is less than 10 mAh. Four sink/source current DACs are applied for nerve stimulation. Each DAC contains four channels, which are programmed using the I^2^C interface. The total output current ranges from 0 to ±1.6mA with 6.3μA steps. The DAC used is Maxim Integrated DS4424, whose output voltage range is 0.5 V to 3.5 V. INL is 1 LSB and DNL is 0.5 LSB. Its output current temperature coefficient is ±75 ppm/°C. The details of component vendors and numbers are shown in Table.1.

The flowchart of the STM32 microcontroller is shown in Fig.5. The amplitude of the current is setup at the initial stage. For non capacitor-coupled biphasic mode, the charge balance could be promised with fine calculation at the beginning, which not require impedance measurement in the later stages. For capacitor-coupled mode, the pulse length will be set to an initial value, and send the pulse to exam the impedance of the electrode and the tissue. After impedance is measured, the pulse width will be recalculated immediately to match the charge balance. The loop happens every 30 minutes.

**FIGURE 3.**
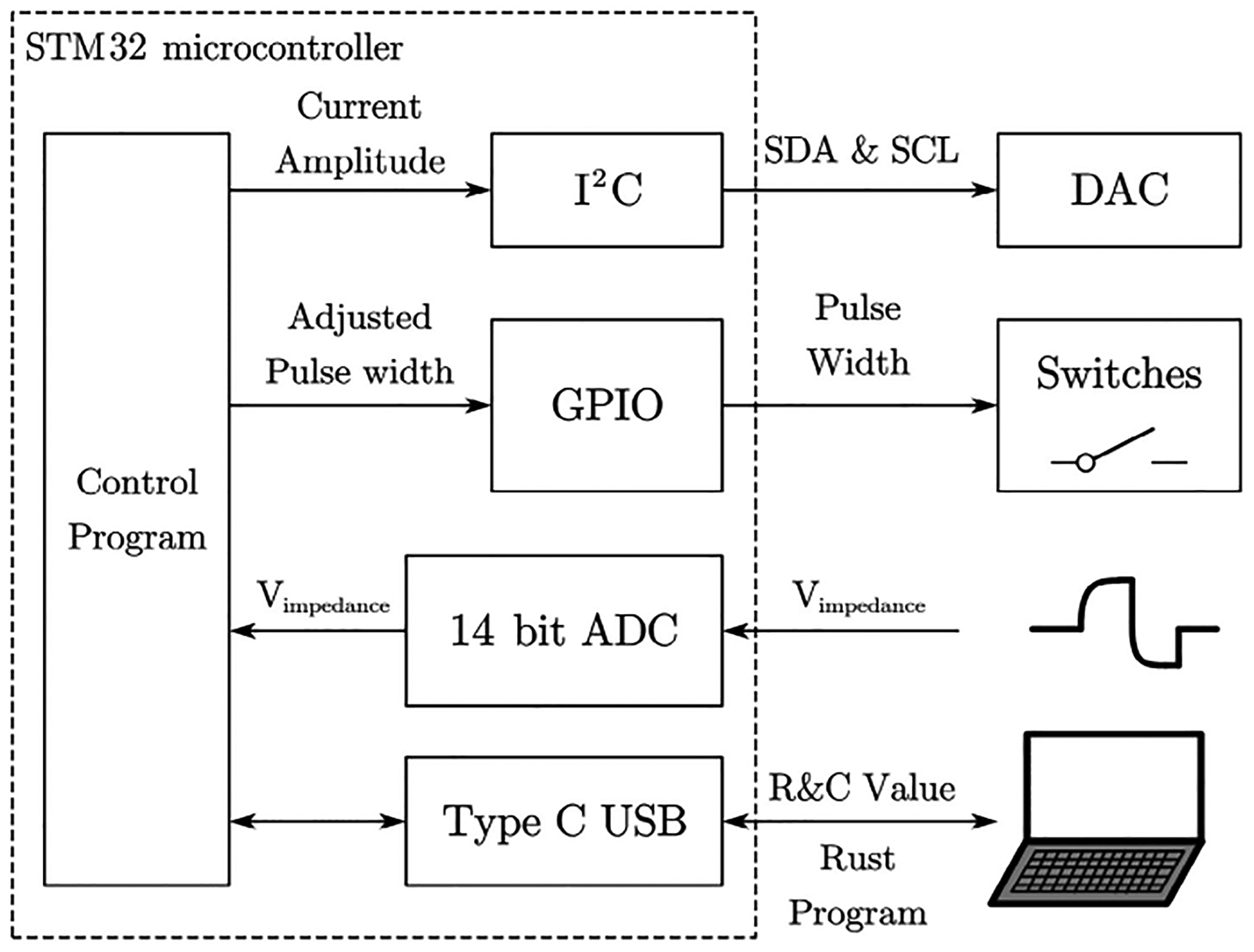
Block diagram of STM32 microcontroller. The DAC is programmed using I^2^C communication. GPIO pins control the switches directly. The impedance voltage will be fed back to the 14-bit ADC. STM32 is programmed by computer through type-C USB. Measured resistor and capacitor value are sent back to the PC for plotting.

**FIGURE 4.**
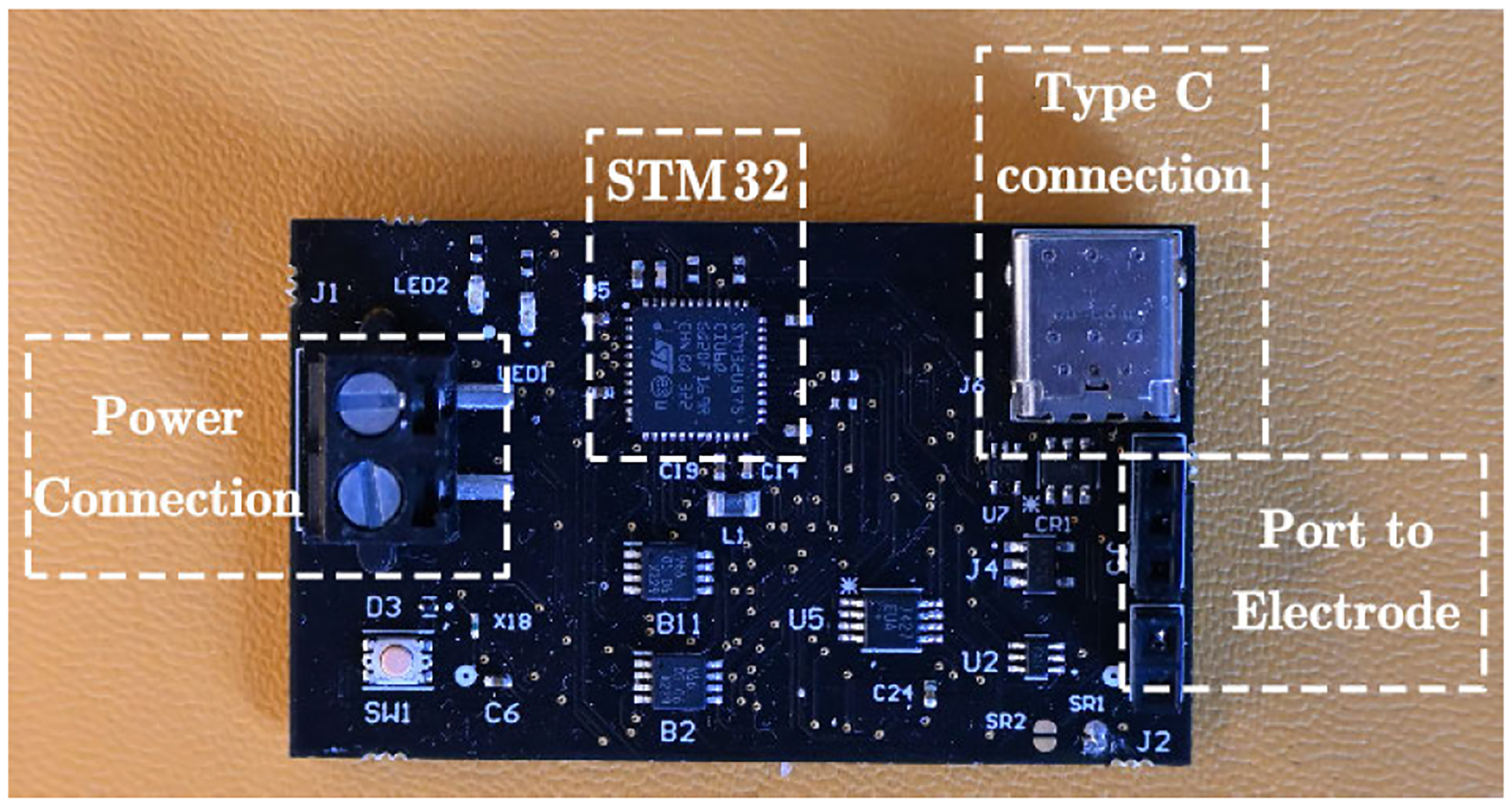
Custom programmable (Type-C port) battery-powered (power port) PCB for analgesic nerve stimulation (electrode port) along with active impedance monitoring unit.

**FIGURE 5.**
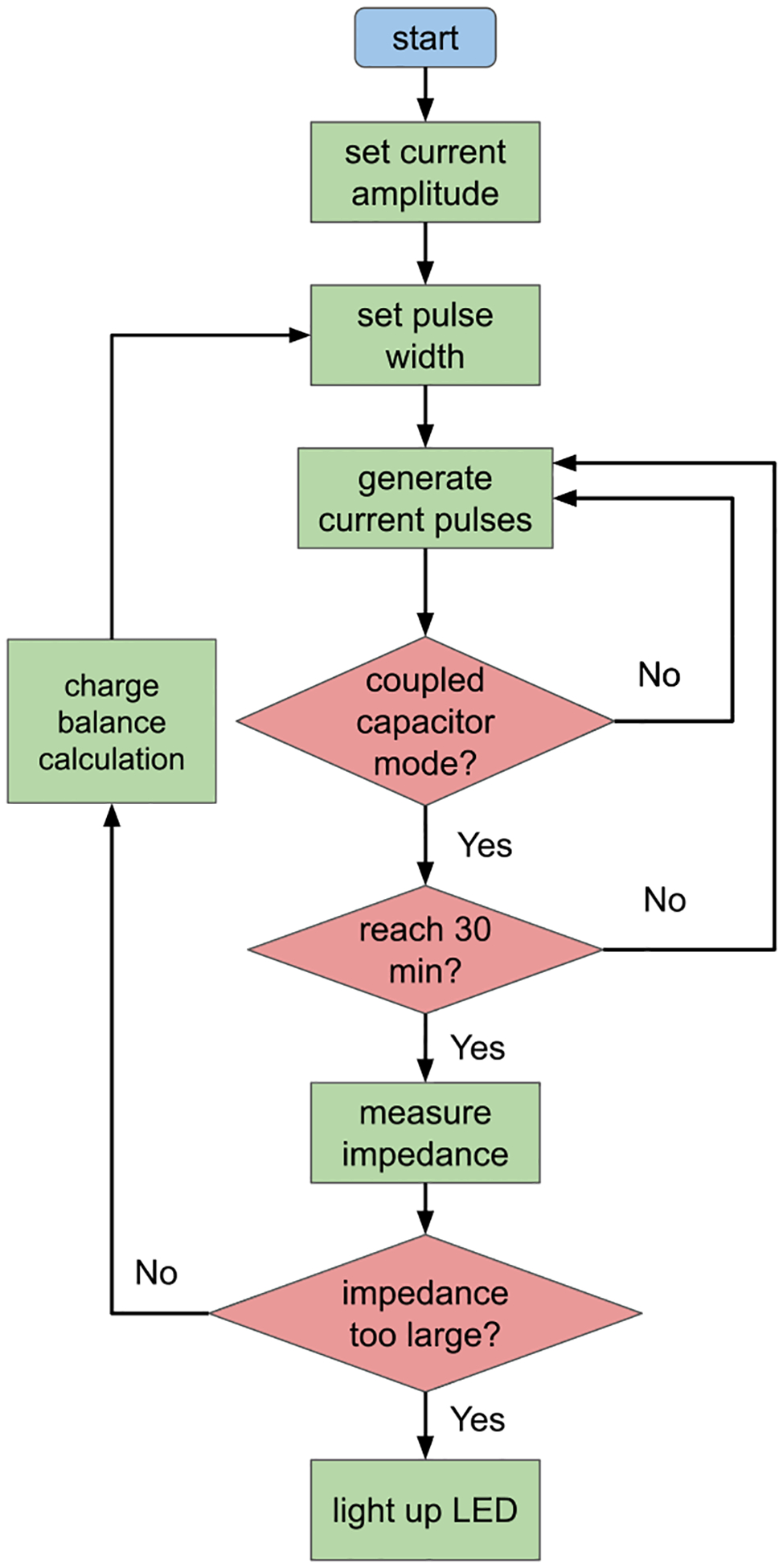
The flow chart of the microcontroller.

For STM32 microcontroller, its block diagram is shown in Fig.3. The program controlled the I^2^C communication to the DAC. GPIO pins are connected directly to the switch and control its on/off statues. For the feedback $V_{\text{impedance}}$, the 14 bits ADC will exam it directly.

From previous studies, the standard stimulation frequency employed for Parkinson’s disease is 130 Hz [28]. For pain relief, alternately 15 Hz and 30 Hz stimulation yielded significant decreases in the severity of pain [29]. In the context of animal testing, high frequencies of stimulation can induce undue distress in animals. It is essential to maintain pulse duration and amplitude within a narrow range to prevent sudden surges in current. Consequently, both medical treatments and animal testing require extended resting intervals coupled with brief activation periods. In contrast, *in vitro* studies benefit from short intervals to facilitate the assessment of electrode impedance. Taking these considerations into account, it is imperative to have a broad frequency range of the stimulation current waveform. This requires greater precision in the digital control logic from the STM32 microcontroller.

To confine the pulse error to within 1μs, the system clock frequency must be configured to a minimum of 16 MHz for the STM32 microcontroller employed in this study. Nevertheless, an elevated system frequency results in increased power consumption, which the coin cell batteries are unable to sustain over extended periods. Consequently, the system clock is segmented into three distinct stages to achieve a balance between precision and energy efficiency.

The initial phase is designated as the system configuration stage, during which the system clock is set to 1 MHz. This phase encompasses the preliminary configuration of all switches, I^2^C communication with current DAC groups, and the initialization and calibration of the ADC. These configurations can be completed using a slow clock. Subsequently, the second phase, known as the operational stage, is initiated. In this phase, the system clock increases to 16 MHz. During this period, a combination of negative and positive pulses is generated, with pulse lengths ranging from μs to several hundred milliseconds. The STM32 facilitates switch control with a precision of μs. Following the operational stage, the process transitions to the resting stage. The duration of the resting stage may vary from a few μs to several seconds, contingent upon the pace of stimulation and the occurrence of an impedance examination. During this phase, no current is transmitted to the electrode. Every 30 minutes, when the system enters the resting stage, an impedance examination is conducted. The STM32 dispatches a series of pulses to construct the Bode plot of the electrode. The impedance degradation of the electrode is a long-term process that takes several days. Measuring the impedance for every stimulation pulse takes too much memory in the STM32 and is not necessary for long term study.

### B. DIGITAL AND ANALOG CONTROL

The entirety of the analog and digital components in the PCB are managed by the STM32 microcontroller. Though STM32 is a commonly used microcontroller, its code could not meet the requirement in this study. Thus the software applications in this study utilize a custom STM32 microcontroller library, which has been internally developed. The program code for STM32 microcontroller is typically generated by STM32CubeMX, which is a user-friendly graphical interface tool provided by STMicroelectronics. The code generated by STM32CubeMX uses a hardware abstract layer (CubeHAL) library to manipulate the hardware. However, the library lacks the essential features required for this work. For instance, CubeHAL’s capability to execute delay functions precisely with an accuracy down to 1 ms. This work needs even finer time control. Additionally, the library does not offer dynamic frequency configuration for power saving. The multi-task feature is supported by the real-time operator system (RTOS), but that there will be loss of precise control of the task.

Thus, a specialized STM32 microcontroller library has been created to enhance the precision of control signal generation. For example, a delay function was implemented to achieve accuracy at the level of 10ns. The custom STM32 microcontroller library facilitates straightforward dynamic updates of both frequency and voltage for the STM32 microcontroller. The frequency of the STM32 microcontroller can dynamically range from 1 MHz to 160 MHz, allowing for fine-tuned management of the system’s power consumption. For high accuracy switch control, the system clock operates at a higher frequency. For general operations, a default frequency of 1 MHz is primarily used to optimize power savings. Rust’s asynchronous feature facilitates the execution of multiple tasks. This capability, inherent in Rust, enables concurrent task execution. Unlike RTOS, asynchronous processing does not interrupt tasks until MCU control is relinquished by the task, allowing for more precise task management.

The custom STM32 microcontroller library also provides functions to manipulate the hardware. It provides Pulse Width Modulation (PWM) functionality to ensure the independence of ADC sampling and stimulation pulses. PWM outputs can be configured on most digital outputs of the STM32, with a duty cycle adjustable between 0.015% to 99.985%. The PWM frequency is dependent on the system clock or other clock source and must be a divisor thereof. Consequently, with a system clock of 1 MHz, the minimum achievable PWM frequency is approximately 15 Hz, which is insufficient for this study. To address this limitation, a prescaler is needed to reduce the frequency. The prescaler allows the PWM clock to be slowed by a whole number factor. A prescaler value is utilized to achieve a PWM frequency range of 1 Hz to 10 kHz.

In this study, the analog-to-digital converter (ADC) of the STM32 microcontroller is used to read the stimulation voltage across the electrod-tissue interface. The sampling rate of ADC is 2.5 Megabits per second with a resolution of 14 bits. The system facilitates both offset and gain compensation. Given that the reference voltage for the electrode-tissue system is not zero, offset compensation is essential to accurately measure the amplitude of the voltage pulses.

As illustrated in Fig. 2, the existing digital-to-analog converters (DACs) engage in communication with the STM32 microcontroller via the I^2^C interface. The amplitude of the current is encoded using an 8-bit format, where the initial bit delineates the sign of the current. The subsequent seven bits determine the amplitude. As illustrated in Fig. 1 (c), four switches are integrated with current DACs and a source capacitor $C_{\text{source}}$. The switches are governed by the STM32 microcontroller, achieving a precision of up to 1μs.

### C. CHARGE BALANCE DESIGN

The electrode-tissue interface can be modeled as a complex impedance with RC circuit elements [24]. The electrode and tissue circuit model is depicted in Fig. 1(c). The resistor $R_{p}$ and capacitor $C_{p}$ represent the tissue impedance, which remains largely unchanged. The resistor $R_{s}$ simulates the impedance of the electrode. During stimulation, the application of a positive pulse results in an accumulation of positive charge, subsequently leading to degradation [27]. With prolonged stimulation, the electrode degrades, causing its impedance to increase significantly. To minimize the effects of positive charge accumulation, a negative pulse must be introduced. When the magnitudes of the positive and negative pulses are balanced, the rate of degradation is reduced. Consequently, regular impedance assessments are essential to appropriately adjust the pulse amplitude and duration.

The proposed system incorporates two distinct modes of stimulation. In each mode, the cumulative negative charge must be balanced by an equivalent positive charge to prevent potential harm to the patient and to prolong the electrode’s operational life. More precisely, the integration of the negative current over time must equal that of the positive current. A prior *in vitro* investigation [27] demonstrated that when utilizing an appropriate waveform, the electrode’s impedance increases by 50Ω after 1 million pulses.

The initial mode under consideration is characterized by the absence of a capacitor coupling. In this mode, the current pulses, both positive and negative, exhibit square waveforms. The intuitive approach proposed to achieve charge equilibrium involves ensuring that the positive area depicted in Fig. 1 (a) is equivalent to the negative area. That is

(1) $$I_{pos}\times t_{pos}=I_{neg}\times t_{neg}$$

The second operational mode is defined by capacitive coupling. In this mode, the negative current waveform assumes a square wave configuration. Conversely, when the current is positive, the source capacitor undergoes charging with a positive current, followed by a subsequent discharge. This process results in the production of an exponential waveform, as depicted in Fig. 1 (b). The resulting smooth exponential waveform is advantageous as it enhances patient comfort and mitigates adverse side effects.

In the capacitor-coupled mode, the onset of the negative pulse coincides with the concurrent charging of the source capacitor. Upon conclusion of the negative pulse, the voltage across the capacitor is

(2) $$V_{\text{capacitor}}=\frac{I_{\text{pos}}}{C_{\text{source}}}t_{\text{charge}}$$

Upon the conclusion of the negative pulse, the capacitor acting as a source initiates discharge, subsequently supplying positive pulses. The discharge current upon the discharge of $C_{\text{source}}$ can be mathematically expressed as

(3) $$I=\frac{V_{\text{capacitor}}}{R_{s}+R_{p}}e^{\frac{-t}{\tau }}$$

where the RC time constant τ could be written as

(4) $$\tau =\frac{R_{s}\times R_{p}\times C_{p}}{R_{s}+R_{p}}$$

To ensure the charge balance, the integration of the discharge current should be equal to the area of the negative current square.

(5) $$I_{\text{neg}}\times t_{\text{neg}}=\frac{V_{\text{capacitor}}}{R_{s}+R_{p}}\int_{0}^{t_{\text{pos}}}e^{\frac{-t}{\tau }}dt$$

Through meticulous calculations, the charge balance can be ensured. References $I_{\text{pos}},t_{\text{pos}}$, and $I_{\text{neg}}$ are assigned constant values, while the negative pulse length $t_{\text{neg}}$ remains variable. This relationship can be expressed as

(6) $$t_{\text{neg}}=\frac{V_{\text{capacitor}}}{\left(R_{s}+R_{p}\right)I_{\text{neg}}}\left(1-\tau e^{\frac{-t_{\text{pos}}}{\tau }}\right)$$

The circuit element values for $R_{s},R_{p}$, and $C_{p}$ are measured through active impedance monitoring.

### D. ACTIVE IMPEDANCE MONITORING

In order to estimate the impedance, the voltage across the electrode and the tissue was measured systematically at 30-minute intervals. As illustrated in Fig. 2, negative current pulses with varying frequencies were applied to the electrode. The measured voltage was acquired by the STM32 ADC using which the frequency response was obtained and plotted on a computer. Before DAC compliance limitation, the range of $R_{s}$ and $R_{p}$ is 16 Ohm to 66kΩ. Using the impedance values at low, high and critical pole frequencies, the values of each circuit component in the electrode-tissue interface model were calculated.

As shown in Fig. 1, in capacitor-coupled biphasic mode, the exponential waveform is proportional to $1/R_{p}+1/R_{s}$. $R_{p}$ represents the tissue resistance, which hardly changed. However, $R_{s}$ represents the electrode resistance, which will change due to electrode degradation over time, body movement, and broken electrodes. This in turn changes the area under the exponential decay, where the negative charge in Fig. 1 is not equal to the positive charge anymore. Thus, active impedance monitoring is needed to adjust $t_{neg}$ in Fig. 1 in order to achieve charge balance. At intervals of 30 minutes, the current stimulator DAC transmits negative current pulses to the electrode to verify its impedance. It is important to acknowledge that the voltage recorded by the ADC encompasses both the real and imaginary components.

(7) $$Z_{\text{measure}}=\frac{V_{\text{measure}}}{I_{\text{stimulation}}}=R_{s}+R_{p}\|\frac{1}{j\omega C_{p}}$$

The equation can be written as

(8) $$Z_{\text{measure}}=R_{s}+\frac{R_{p}}{1+\omega^{2}{R_{p}}^{2}{C_{p}}^{2}}-j\frac{\omega {R_{p}}^{2}C_{p}}{1+\omega^{2}{R_{p}}^{2}{C_{p}}^{2}}$$

With precise tuning of the pulse frequency, the value of the resistances and capacitance of the electrode-tissue interface impedance can be calculated according to the frequency response.

In scenarios where the frequency is significantly below the cutoff frequency, denoted as ω→0, the impedance can be expressed as $Z=R_{s}+R_{p}$. Conversely, when the frequency surpasses the cut-off frequency, indicated as ω→∞, the impedance is simplified to $Z=R_{S}$. When the stimulation frequency is close to the pole frequency, i.e. ω is close to $\frac{1}{R_{p}C_{p}}$, the measured impedance will be $Z=Rs+R_{p}/2$. Therefore, the precise determination of the cut-off frequency serves useful for calculating the capacitance $C_{p}$.

It should be noted that the ADC-sampled data comprise both real and imaginary components, as the magnitude of impedance depicted in Equation (9). At extremely low or high frequencies, the imaginary component approaches zero. Conversely, when $\omega =1/R_{p}C_{p}$, the amplitude of the imaginary component reaches $\frac{R_{p}}{2}$ and the measured impedance will be

(9) $$Z_{\text{cutoff}}=\sqrt{{\left(\frac{R_{p}}{2}\right)}^{2}+{\left(R_{s}+\frac{R_{p}}{2}\right)}^{2}}$$

which is significantly larger than $R_{s}+R_{p}/2$. Therefore, to obtain precise results, all measured data must be corrected by subtracting the imaginary component. In the STM32 microcontroller, the ADC requires approximately 5μs for sampling when the system clock is operating at 1 MHz. To ensure that the ADC sampling phase does not coincide with the current pulse, the switches are transitioned to PWM mode during the impedance measurement stage. The duty cycle is maintained at a constant 50%. From a range of 1 Hz to 10 kHz, eight distinct frequencies are sampled per decade. For each frequency, the ADC continuously samples 1000 data points to calculate the resulting voltage drop between them.

Given that there are only 8 frequency responses per decade, the precise cut-off frequency may not be present among these data points. To address this issue, the three impedance found in proximity to the equation (10) will be selected. Their corresponding frequency will be used for linear regression, from which the exact cut-off frequency $f_{\text{cutoff}}$ will be derived.

Then the capacitor $C_{p}$ value can be written as

(10) $$C_{p}=\frac{1}{2\pi R_{p}f_{\text{cutoff}}}$$

Based on the frequency response, the real part of each $Z_{\text{measure}}$ is,

(11) $$Z_{\text{real}}=\sqrt{{Z_{\text{measure}}}^{2}-\left(\frac{{2\pi f{R_{p}}^{2}C_{p}}^{2}}{1+4\pi^{2}f^{2}{R_{p}}^{2}{C_{p}}^{2}}\right)}$$

With appropriate error correction, the measured impedance is closer to the ideal value. The results of error corrected measurements of impedance values are presented in Section IV. Given that the operational voltage for the entire system is within the range of 0 to 3.3 V, an increase in electrode impedance to elevated levels results in the measured voltage $V_{\text{measure}}$ surpassing the operational voltage range. In such cases, an indicator LED will be activated to notify the user.

The stimulator in this work has some limitations. The stimulator maximum current is ±1.6 mA. For some *in vitro* study and animal study, this value may not be large enough. Also, the stimulator connection to the computer is through a Type C USB cable. It limits the movement of the testing animal when measuring impedance. Wireless connection would be preferred in this case. These limitation will be further discussed in Section IV.

## III. RESULTS

The stimulator PCB was validated by employing two distinct modes. As illustrated in Fig. 2, the PCB was configured for *in vitro* studies utilizing a two-electrode arrangement immersed in a solution of buffered artificial cerebrospinal fluid (aCSF, pH 7.4). The PCB was employed in an extended-duration study for continuous current stimulation and impedance monitoring to eventually perform charge balancing. The verification of the impedance feedback was conducted initially using an discrete RC circuit, as depicted in Fig. 1. The electrode is modeled with a simple resistor $R_{s}$. Elements $R_{p}$ and $C_{p}$, connected in parallel, model the buffered artificial cerebrospinal fluid solution.

### A. CURRENT STIMULATION

In order to intuitively represent the current waveform, the stimulation was applied to an ideal resistor, with the resultant voltage subsequently measured. The current was then calculated in accordance with Ohm’s law where $I=\frac{V}{R}$.

**FIGURE 6.**
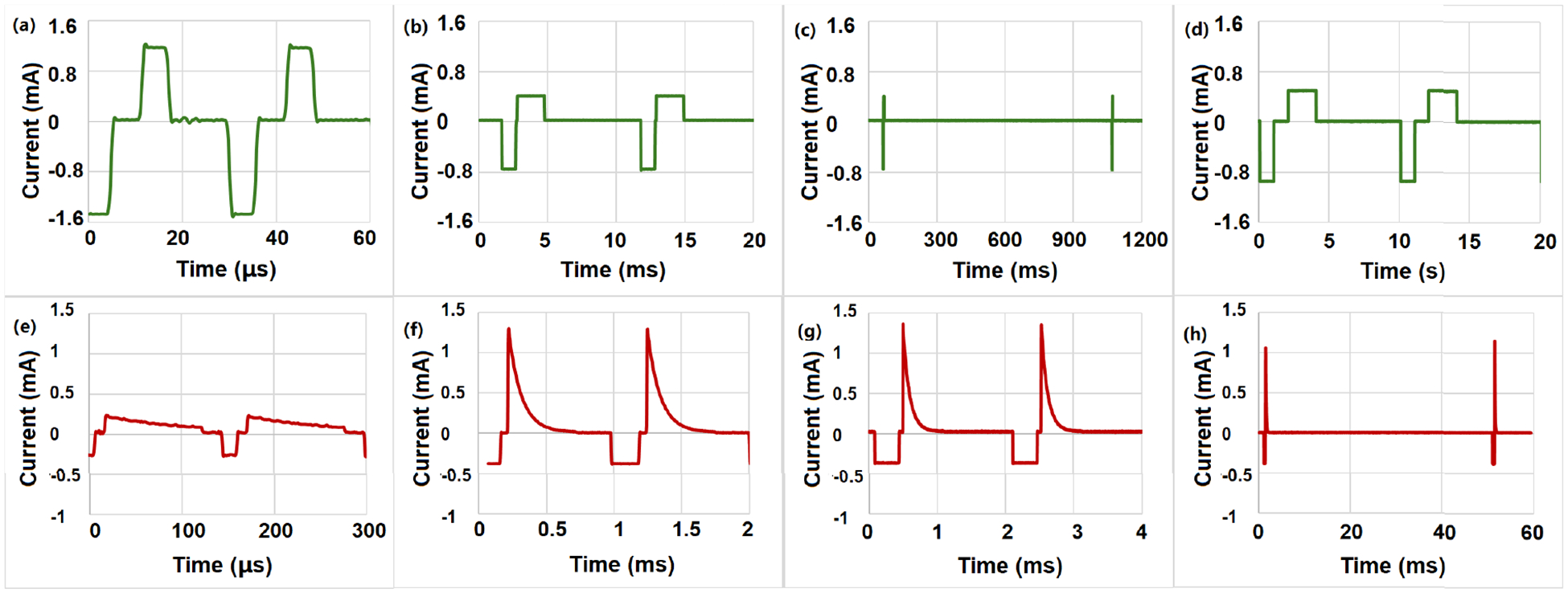
Measured stimulation current waveforms for two modes with different amplitude and frequency. The first row is non-capacitor coupled mode, and the second row is the capacitor coupled mode. Subfigure (b),(c),(d) are charge balanced. **A** $I_{\text{pos}}=1.2\;\text{mA},I_{\text{neg}}=-1.5\;\text{mA},t_{\text{pos}}=t_{\text{neg}}=5\;\mu \text{s}$, Total period is 30μs. **B** $I_{\text{pos}}=0.4\;\text{mA},I_{\text{neg}}=-0.8\;\text{mA},t_{\text{pos}}=2\;\text{ms},t_{\text{neg}}=1\;\text{ms}$, Total period is 10 *ms*. **C** $I_{\text{pos}}=0.4\;\text{mA},I_{\text{neg}}=-0.8\;\text{mA},t_{\text{pos}}=2\;\text{ms},t_{\text{neg}}=1\;\text{ms}$, Total period is 1 s. **D** $I_{\text{pos}}=0.5\;\text{mA},I_{\text{neg}}=-1.0\;\text{mA},t_{\text{pos}}=2\;\text{s},t_{\text{neg}}=1\;\text{s}$, Total period is 10 s. **E** $I_{\text{pos}}=0.2\;\text{mA},I_{\text{neg}}=-0.2\;\text{mA},t_{\text{pos}}=100\;\mu \text{s},t_{\text{neg}}=20\;\mu \text{s}$, Total period is 130μs. **F** $I_{\text{pos}}=1.3\;\text{mA},I_{\text{neg}}=-0.4\;\text{mA},t_{\text{pos}}=500\;\mu \text{s},t_{\text{neg}}=200\mu \text{s}$, Total period is 1 ms. **G** $I_{\text{pos}}=1.3\;\text{mA},I_{\text{neg}}=-0.4\;\text{mA},t_{\text{pos}}=600\;\mu \text{s},t_{\text{neg}}=350\;\mu \text{s}$, Total period is 2 ms. **H** $I_{\text{pos}}=1.1\;\text{mA},I_{\text{neg}}=-0.4\;\text{mA},t_{\text{pos}}=600\;\mu \text{s},t_{\text{neg}}=240\;\mu \text{s}$, Total period is 50 ms.

Figure 6 illustrates the current through a 1,000Ω resistor subjected to two distinct stimulation modes. For Fig. 6, 4 different pulse width and duty cycle are presented for both stimulation modes and charge balance is not performed. For biphasic capacitor-coupled mode, the maximum current is larger than 1.6 mA because it is generated by capacitor discharge. This large transient current has a limitation due to limited working voltage range and will be helpful in reversing some of the desired physiological effects of the first phase [15]. The first row represents a non-capacitor coupled mode, whereas the second row depicts a capacitor coupled mode. The total stimulation duration varies between 30μs and 10 s. Pulse lengths are adjustable from 5μs to 2 s. The stimulator can accommodate both extended resting intervals and brief working phases.

### B. IMPEDANCE MEASUREMENT

The STM32 microcontroller sends negative current pulses to the electrode every 30 minutes to determine impedance. The pulse frequency ranges from 1 Hz to 10 kHz, with eight distinct frequencies per decade, effectively encompassing the typical range of variations observed in the electrode. In order to facilitate observation, we connect the STM32 to the computer to display the impedance data.

Two discrete RC circuit groups are employed to simulate the electrode and tissue characteristics. The corresponding circuit model is illustrated in Fig. 1, and the frequency response is depicted in Fig. 8. In subfigure (a), the values for $R_{s},R_{p}$, and $C_{p}$ are specified as 220Ω,510Ω, and 2.2μF, respectively. These values are based on Autolab measurement of electrode-solution system. The capacitance value for $C_{p}$ is subject to change when the electrode is immersed in a different solution. To replicate this scenario, in subfigure (a), the values for $R_{s}$ and $R_{p}$ remain consistent with those in subfigure (a); however, $C_{p}$ is set to 2.2μF. Additionally, when the electrode undergoes degradation, $R_{s}$ increases from several hundred Ohms to thousands of Ohms. For the purpose of evaluating the accuracy of the PCB measurement, the value of $R_{s}$ is adjusted to 510Ω, while all other conditions are maintained as in subfigure (a).

For comparative analysis, an ideal setup is modeled in MATLAB, and the simulation results are depicted in Figure 8. It is evident that PCB measurements consistently exceed the true value regardless of frequency. Due to the ADC sampling error, there exists a constant error of 30Ω. This error was subsequently rectified by subtracting the imaginary component after which the measured data was replotted. In Fig. 8(a), the purple line represents the adjusted Bode plot. The computed values for $R_{s},R_{p}$, and $C_{p}$ are 218.16Ω,517.02Ω, and 2.13μF, respectively. In sub-figure (b), the computed values for $R_{s},R_{p}$, and $C_{p}$ are 222.65Ω,507.59Ω, and 4.61μF. In sub-figure (c), the calculated values for $R_{s},R_{p}$, and $C_{p}$ are 511.95Ω,505.91Ω, and 2.14μF. Across all three test cases, the error is reduced to under 10% following the adjustments. When measurement frequency is very high, the error is dominated by the high frequency noise.

**FIGURE 7.**
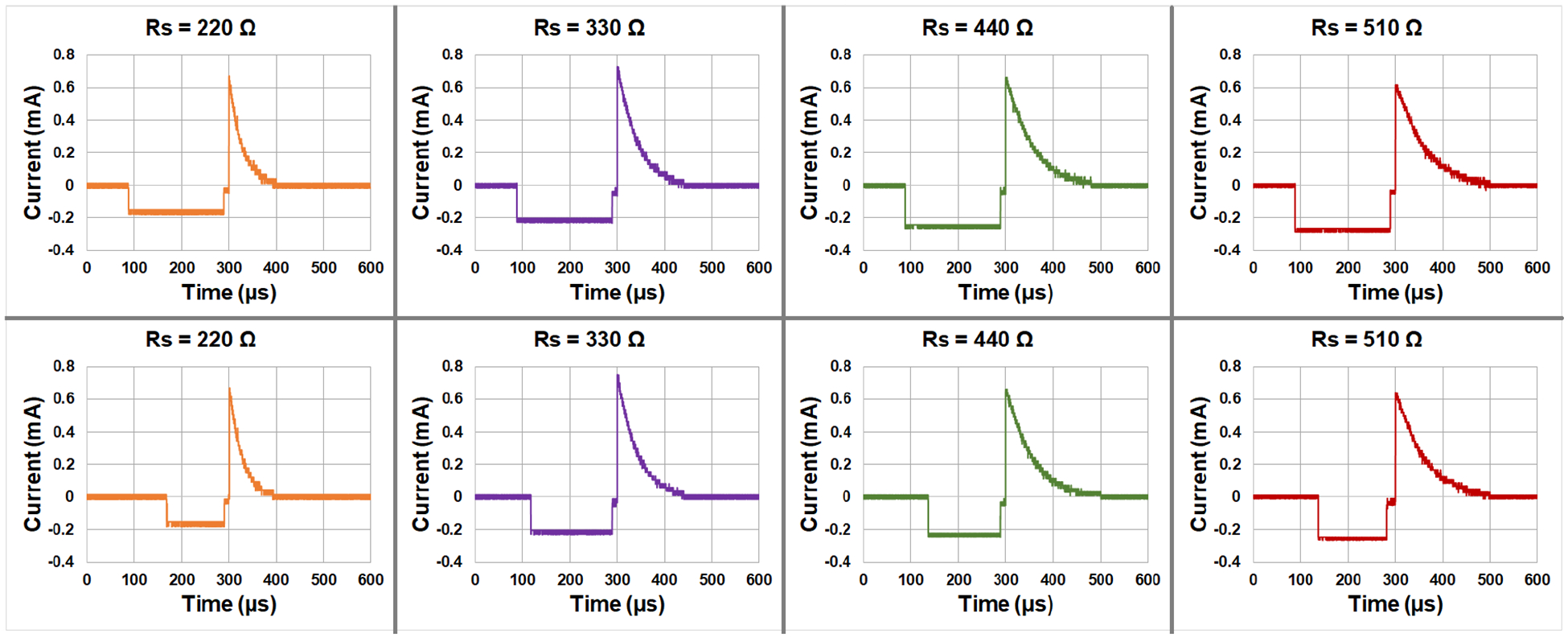
Measured stimulation current waveforms for RC circuits with increasing $R_{s}$ value to emulate electrode degradation. The first row is before charge balance correction and the second row is after charge balance correction illustrating area equivalency between positive and negative phases. The resistor $R_{p}$ is 510Ω and the capacitor $C_{p}$ is 22μF. The resistor $R_{S}$ increases from 220Ω to 510Ω.

**FIGURE 8.**
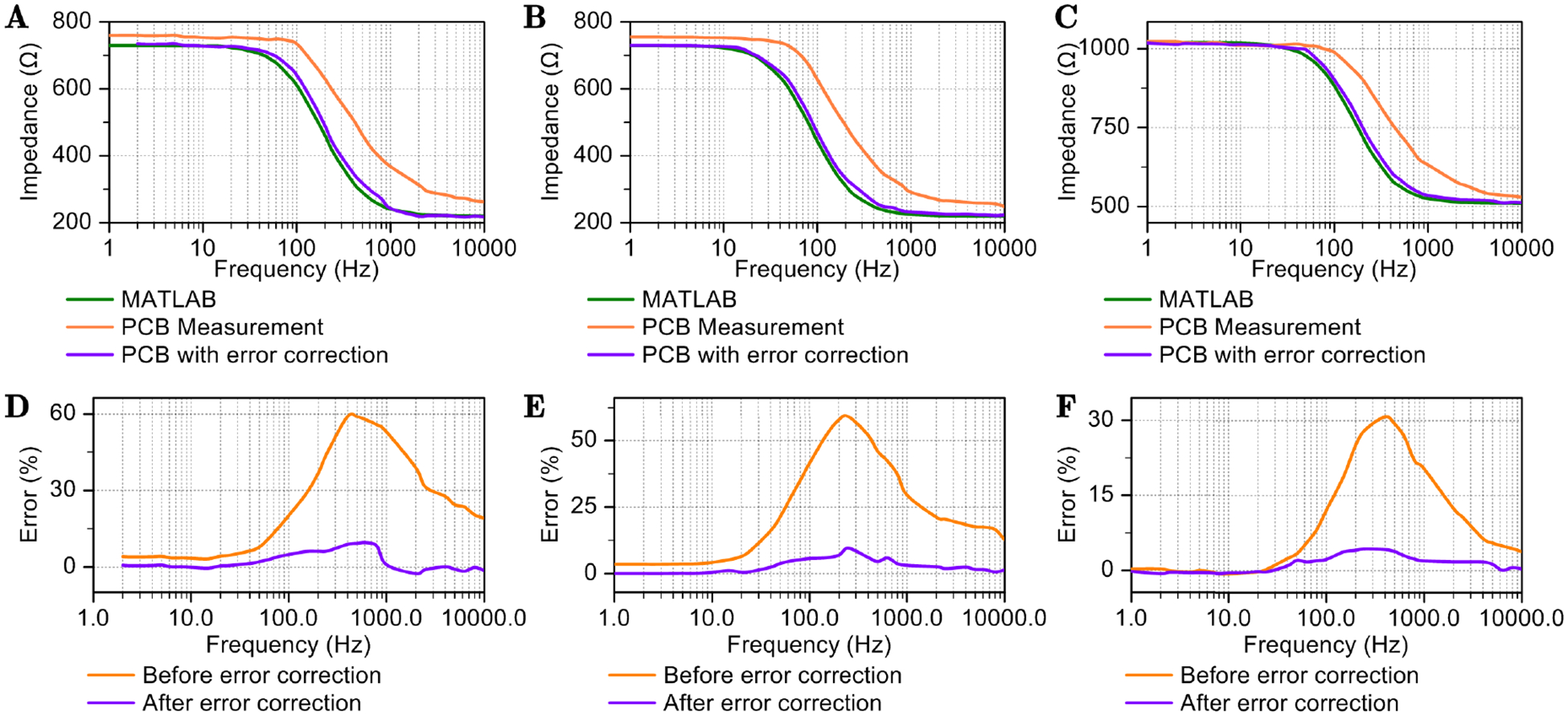
Bode plots obtained via PCB measurements and the associated errors are presented. The first row illustrates Bode plots for various RC circuits. The green line represents the outcomes of MATLAB simulations, the orange line denotes the results from PCB measurements, and the purple line depicts measurement results subsequent to error correction. The second row displays the corresponding errors in PCB measurements, both prior to and following error correction. A $R_{S}=220\;\Omega .R_{p}=510\;\Omega .C_{p}=2.2\;\mu \text{F}$. B $R_{S}=220\;\Omega .R_{p}=510\;\Omega .C_{p}=4.7\;\mu \text{F}$. C $R_{S}=510\;\Omega .R_{p}=510\;\Omega .C_{p}=2.2\;\mu \text{F}$.

In the context of the capacitor-coupled mode, the effect of charge balancing needs to be meticulously derived. Figure 7 illustrates the voltage behavior under current stimulation in this mode. The parameters $R_{p}$ and $C_{p}$ are configured with values of 510Ω and 22μF, respectively. These values are selected according to the measurement result of the electrode-tissue impedance from the buffer solution, measured using Autolab. Both the negative current and the positive charge current amplitude are set to 0.5mA. As the electrode undergoes degradation, an increase in impedance is expected to be observed. To emulate this phenomenon, the parameter $R_{s}$ is assigned the values 220Ω,330Ω,440Ω, and 510Ω, respectively. Figure 7 illustrates the alterations in the voltage waveform with electrode degradation. The first column presents the unbalanced charge waveform with a default negative and positive pulse duration set at 200μs. The second column depicts the corresponding balanced waveform. While the positive area remains consistent across each column, the negative area is modified in the second row, with negative pulse durations set at 136μs,141μs,148μs, and 151μs for each respective column.

As outlined in the preceding section, a balanced charge implies that the positive and negative regions of each curve must exhibit area equivalency. To ascertain that charge is balanced, the areas corresponding to each segment of Figure 7 are presented in Table 2. The demarcation created by the grid lines is 40 nC. Based on the measured impedance, the deviation between the positive and negative charges is determined to be less than 5%.

### C. IN VITRO STUDY

In our prior research work [27], it has been demonstrated that employing a charge balanced biphasic waveform resulted in an electrode impedance increment of merely 5Ω after the delivery of 4 million pulses. Consequently, a comparative analysis of the biphasic waveform will be omitted in this study. This work will concentrate on the charge balanced capacitor-coupled biphasic mode, attributing to the intricate nature of its charge calculation.

**FIGURE 9.**
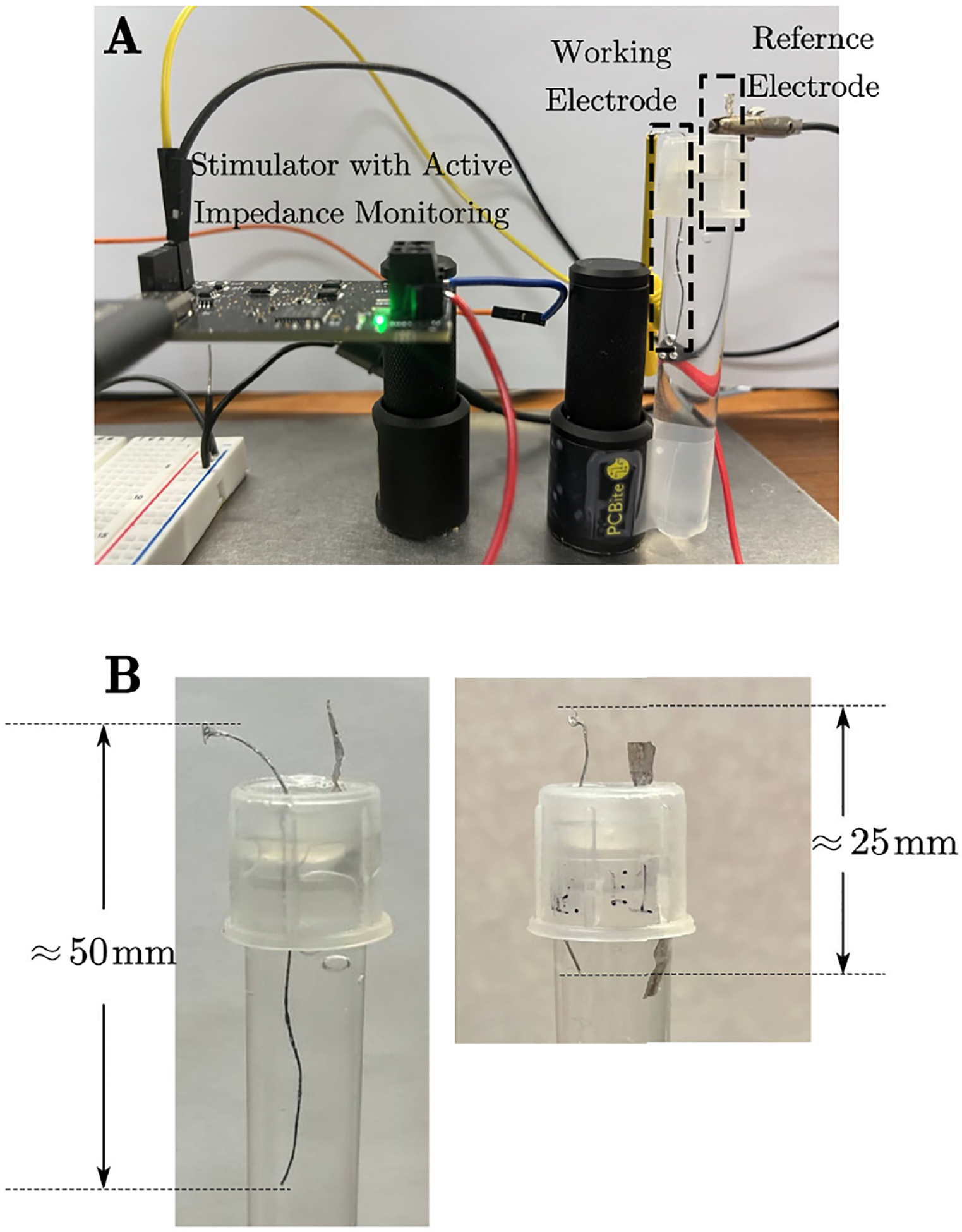
**A** Set up of *in vitro* study. Stimulator is powered by 5V DC power supply. The working electrode is connected to the stimulator output. The reference electrode is connected to the 3.3V DC voltage. Both electrodes are soaking in the buffered artificial cerebrospinal fluid to mimic animal tissue. **B** Electrode photo after long period stimulation. Left: Electrode stimulated for 80 hours with charge balance correction. The electrode’s initial length is approximately 50 mm, and it is maintained in good shape. Right: The electrode stimulates for 30 hours without charge balance correction. The electrode breaks due to degradation. The left length is only 25 mm.

The capacitor-coupled modes were assessed *in vitro* employing a two-electrode configuration in a buffered artificial cerebrospinal fluid (aCSF, pH 7.4) solution, as illustrated in Fig. 9. The working electrode consisted of an insulated degradable metal wire with a 1 cm exposed tip. The electrode is made of 99% zinc. The wires are 100μm each and 4 strands. The geometric surface area is $12.56\;{mm}^{2}$. The insulation material is polyurethane and it is 50μm thick. The wire is manually coiled then dipcoated in the 15% polyurethane solution in hexafluoro isopropanol. In most cases, the charge density of this electrode is $1.6\mu C/cm^{2}$, which is in the common $30\mu C/cm^{2}$ safety limit. The electrode is not intentionally made more fragile even though it is bioresorbable. The use of the zinc metal over stronger materials such as stainless steel does result in a device with decreased mechanical strength, but this is a consequence of the material choice. Since these devices are intended to have a useful lifetime of 60 days, and due to other factors in their use such as the shallow placement of the electrode in the tissue and the other real world mitigation that can be employed, this was not seen as a major detriment. The quality of the electrodes was measured predominantly through visual inspection on an optical microscope and EIS. Once reproducibility was consistently achieved, the use of EIS enabled confirmation of the conductivity and area of the electrode site, as well as confirmation of the insulation integrity.

A $4\times 20\;{mm}^{2}$ platinum foil was used as the counter electrode. A 3.3 V DC voltage is connected to the counter electrode to set the voltage measuring baseline. The voltage across the electrode was measured every 30 minutes using an STM32 microcontroller to evaluate alterations in the working electrode resistance, indicative of degradation. The impedance data provides a clear indication of electrode degradation. The LED light on the PCB turns on when the impedance of the electrode is too large.

The initial negative phase is characterized by an amplitude of 0.5 mA and possesses an adjustable pulse width. This phase is succeeded by a positive, capacitor-coupled phase. The positive phase is facilitated by a 0.1μF capacitor on the PCB, which is charged with a 0.5 mA current over a duration of 150μs, and it discharges over a time span of 200μs. These waveform characteristics were carefully chosen due to their clinical significance. The negative phase serves the purpose of providing analgesic stimulation, whereas the positive phase ensures charge balancing. Mitigating the amplitude of the positive phase through the implementation of asymmetry or capacitor coupling is advantageous in terms of reducing electrode degradation.

**FIGURE 10.**
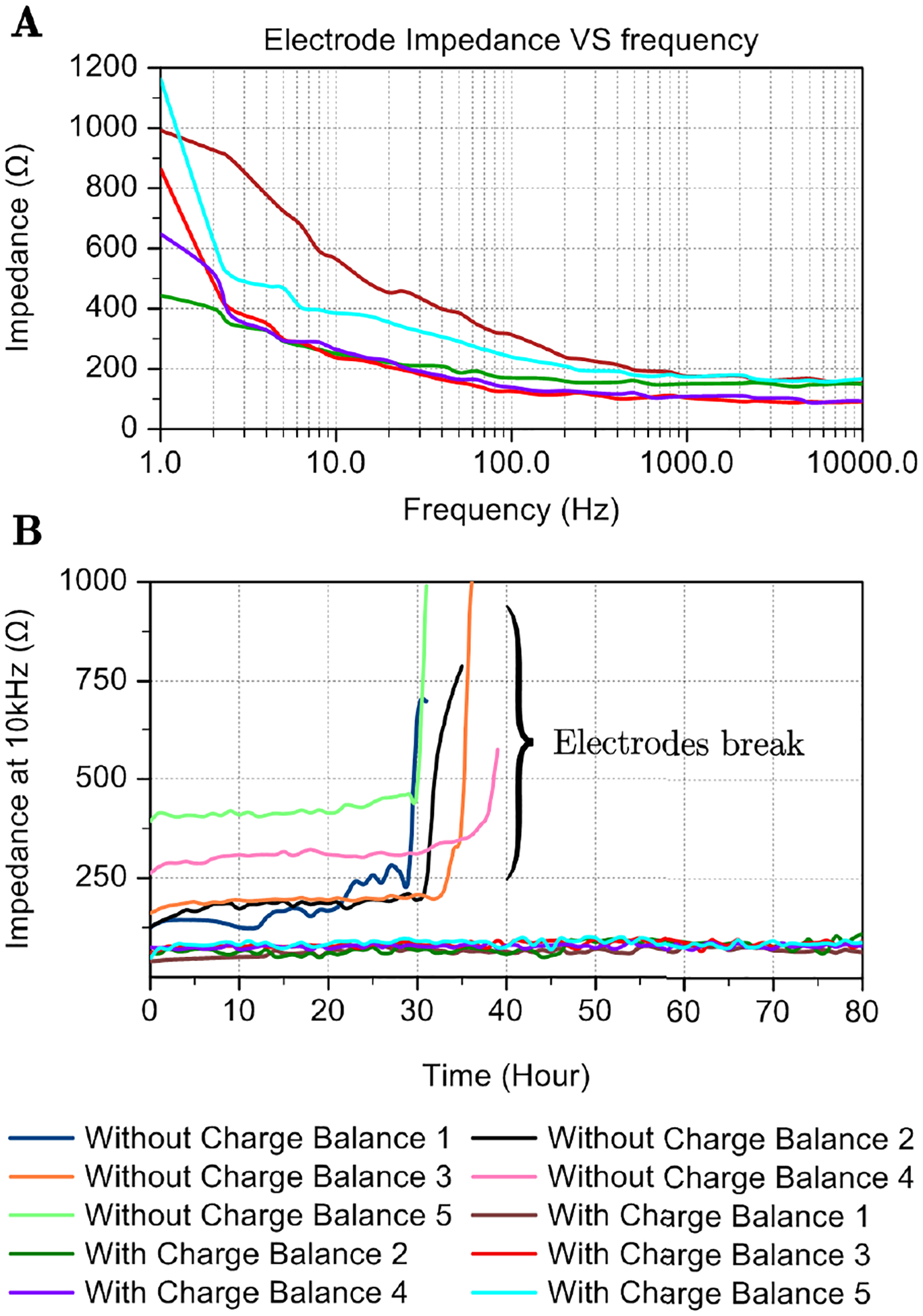
**a** Frequency response of 5 different electrode-solution systems measured *in vitro* using the active impedance monitoring unit in the PCB. **b** Measured electrode resistance $R_{S}$ change with time. There are 2 groups of electrodes with 5 electrodes per group that wee stimulated. Both groups are stimulated at 1 kHz in capacitor-coupled mode. The first group is stimulated without charge balance correction and the second group performs charge balance every 30 minutes. The unbalanced electrodes break after 30 hours of stimulation. For the group with charge balancing, the impedance change is less than 20Ω after 80 hours of stimulation.

To show the initial condition of electrodes-solution system, five groups of electrodes-solution system impedance are measured, as shown in Fig.10. The frequency ranges from 1 Hz up to 10 kHz. Since we treat the system as RC circuit shown in Fig. 1, at 10 kHz the impedance value could be treated as the impedance of electrode only. It could be observed that the all five electrode impedance ranges from 100Ω to 200Ω.

Fig. 10 shows the change in electrode resistance $R_{S}$ with time for 2 groups of electrodes. The first group has 4 electrodes that are stimulated without charge balance correction. For the second group with 5 electrodes, charge balancing is implemented based on the resistance measured (impedance at 10 kHz), and the stimulation pulse is adjusted every 30 minutes. The stimulation frequency for both electrodes is 1 kHz. Due to the differences in electrode shape and counter electrode position, the initial resistance of these 2 groups is different. For the first 30 hours, the resistance of electrodes without charge balance kept increasing. Then, the electrode resistance jumped abruptly to more than 600Ω, indicating that the electrodes broke. For the 5 electrodes that broke in the *in vitro* test, they worked 28, 29.5, 30.5, 32, 38 hours respectively. In average, they worked 31.7 hours. Looking at the electrodes, it was evident that the degradation resulted in an exposed tip fracture, as shown in Fig. 9 right. The initial length of the electrodes is about 50 mm. After they break, there are only 25 mm left. However, the resistance of 5 electrodes with charge balance barely increases, even after 80 hours of continuous stimulation. For all 5 electrodes, their resistance increase by less than 20Ω. In average, the lifetime is improved 2.52 times. As shown in Fig. 9, the left one is the photo of the electrode after 80 hours of stimulation with charge balancing. The electrode length is the same as the initial state. There is no obvious break point on the exposed part of the electrode. The results clearly demonstrate that using capacitor-coupled stimulation with online charge balancing through active impedance monitoring enhances electrode stability compared to stimulation scenarios lacking charge balance calculation.

## IV. CONCLUSION AND DISCUSSION

This study presents the design and implementation of a stimulator for chronic pain relief. The stimulator treats patients by connecting metal electrodes, which is implanted to the human tissue. The PCB remains outside the body. It features two operational modes: biphasic with and without capacitor coupling. All operation modes could be custom defined with different stimulation pulse amplitudes and lengths. All stimulated pulses are current-controlled and could be used in charge balance calculation directly.

The stimulator is implemented on a 30 × 50 mm PCB, to achieve a small factor to fit on the backpack for future animal studies. The stimulator offers a programmable stimulation range encompassing a current of ±1.6 mA with 6.3μA step.

The custom STM32 microcontroller library offers a temporary high-speed system clock with precision in 100 ns. It enables the pulse width tuning from 1μs to 10 seconds in μs scale with overall low power consumption. The stimulation frequency for both operation modes could be set from 0.1 Hz to 10 kHz with carefully calculated pulse width and amplitude.

Electrical testing reveals that the stimulator accurately registers resistance variations in the electrode-tissue interface within a margin of error 10% in both modes, as shown in Table 2. In a consistent-current biphasic mode, charge balancing between the positive and negative currents could be achieved by calculation of the current amplitude and period. Its mathematical reference is shown in eq.II-C. For biphasic capacitor-coupled mode, with impedance measurement feedback, the charge discrepancy noted to be less than 5%. The DAC current error is 0.2% while the STM32 microcontroller timing error is 0.25%. Compared to the total error, these two errors are negligible.

Without charge balancing, the degradation of the working electrode from 120Ω to 660Ω at 1 kHz stimulation over a testing period of 30 hours in biphasic mode *in vitro* is documented. In contrast, the capacitor-coupled mode with charge balance exhibits adequate electrode stability, enduring up to 288 million pulses with only 25Ω change in electrode resistance, as shown in Fig. 10. The *in vitro* studies clearly demonstrated the efficacy of charge-balanced stimulation that allows electrodes to last approximately 3 times longer with negligible degradation. It shows potential to sustain the lifetime of the mental electrode in a long-term animal study.

The comparison with other recent work on programmable stimulators is shown in Table 3. The power consumption is measured with biphasic stimulation mode. The frequency of stimulation is 100 Hz with 10% duty cycle and the STM32 microcontroller system clock is 1 MHz. Supply power is 5 V. When working in capacitor-coupled biphasic mode, the total power consumption remains the same. Power consumption will not increase with stimulation frequency, duty cycle or operational mode, since the total current consumed by the DACs remains the same. Power will increase linearly with supply voltage since P=VI and marginally increase with MCU clock frequency which affects the dynamic power. It is evident that this work is unique because it incorporates impedance monitoring and online charge balancing to slow down electrode degradation. Compared with other state-of-the-art works, this work shows multiple choices on stimulation modes and stimulation frequency. The custom STM32 microcontroller allows for 1μs scale changes in both stimulation pulse and total period.

Future work involves using the PCB system for animal studies with long-term stimulation and impedance monitoring. The power consumption can be reduced further with a custom analog integrated circuit. The stimulator aims to continue working for more than 24 hours in animal testing. Also, future work involves storage and transfer of measured impedance data. Large amount of impedance data can be stored in STM32 microcontroller and transferred on demand. This reduces the constraints on the animal experiments resulting in easier operation.

## Figures and Tables

**TABLE 1.** Components vendor and number.

Component	Vendor	Part Number
Current DAC	Maxim Integrated	DS4424
Microcontroller	STMicroelectronics	STM32U5A5
Power Regulator	Diodes Incorporated	AP62200WU
Analog Switch	Nexperia USA Inc.	74LVC2G66DP

**TABLE 2.** Charge balance error calculation for capacitor-coupled biphasic mode.

$R_{s}(\Omega )$	220	330	440	510
Positive Area (nC)	80	140	170	176
Unbalanced Negative Area (nC)	120	180	220	240
Unbalanced Error (%)	50.00	28.57	29.41	36.36
Balanced Negative Area (nC)	84	144.8	175.2	182
Balanced Error (%)	5.00	3.42	3.06	3.41

**TABLE 3.** Stimulator performance comparison Table.

	TCAS [30]	TBioCAS [31]	Electronics [32]	PRIME [33]	TBioCAS [34]	This work
Average Power per Channel [W]	N/A	2.6m	N/A	N/A	38.61m	5.65m
Stimulation Modes	Biphasic	Biphasic	Biphasic	Biphasic	Biphasic	Monophasic, Biphasic, Cap-Coupled Biphasic
Supply Voltage [V]	9	1.3,3.3	±20	3 – 9	±6	5 – 12
Channels	8	1	8	2	16	2
Stimulation Frequency Range [Hz]	100k	15,50	100 – 1.2M	392 – 50k	1 – 100k	0.1 – 10k
Maximum Stimulation Current [A]	70μ	750μ	±1m	±10m	±3m	±1.6m
Stimulation Steps [μA]	10	20	3.9	16.11	200	6.3
Impedance Monitoring	No	No	No	No	No	Yes
